# Koch’s Remedy, in Relation to Throat Consumption

**Published:** 1891-06

**Authors:** 


TREATMENT OF THE "RED NOSE."—A red nose is a very painful affection, especiatly when it attacks females. According to Unna, one-fifth of the cases are due to acne rosacea with vascular dilatation. Very often it stands in direct relation to seborrhea of the hairy skin. This seborrhea should be treated in the usual way. When acne rosacea is the cause, Unna gives fifty centigrams (seven and a half grams) of ichthyol daily internally, and at the same time prescribes lotions of the same substance in watery solution externally. At night, applications of the following paste are of benefit:

| | |
|---|---|
| Zinc pomade, - - - | 20.0 |
| Rice powder, - - - | 5.0 |
| Sulphur, - - - - | 2.0 |

Unna advises multiple scarifications of the dilated veins after Hebra. This should be repeated two or three times a week. The minute wounds should be covered at once with moist absorbent cotton. In light cases, and as supplementary treatment, he advises repeated washings with ichthyol soap. Only warm water should be used.—*Amer. Pract. and News*, May, 1891.

